# The Evolution of Genetic Variability at the *LRRK2* Locus

**DOI:** 10.3390/genes15070878

**Published:** 2024-07-03

**Authors:** Dylan T. Guenther, Jordan Follett, Rim Amouri, Samia Ben Sassi, Faycel Hentati, Matthew J. Farrer

**Affiliations:** 1 Department of Neurology, University of Florida, Gainesville, FL 32610, USA; 2 Mongi Ben Hamida National Institute of Neurology, Av. de la Rabta, Tunis 1007, Tunisia

**Keywords:** *LRRK2*, Parkinson’s disease, evolution

## Abstract

Leucine-rich repeat kinase 2 (*LRRK2*) c.6055G>A (p.G2019S) is a frequent cause of Parkinson’s disease (PD), accounting for >30% of Tunisian Arab-Berber patients. *LRRK2* is widely expressed in the immune system and its kinase activity confers a survival advantage against infection in animal models. Here, we assess haplotype variability *in cis* and *in trans* of the *LRRK2* c.6055G>A mutation, define the age of the pathogenic allele, explore its relationship to the age of disease onset (AOO), and provide evidence for its positive selection.

## 1. Introduction

Globally, genetic variability within *LRRK2* confers the highest genotype- and population-attributable risk for PD [1], and the frequency of many variants appears to be population-specific; *LRRK2* p.R1441G/C is most frequent in European Basques [1] and Belgians [2]/Italians [3], respectively, p.G2385R [4] and p.R1628P [5] are found in East Asians, and p.G2019S is common in Ashkenazi Jews [6] and North African Arab-Berbers [7]. Notably, all pathogenic *LRRK2* mutations elevate its kinase activity, whereas p.G2019S (rs34637584_A) directly breaks the hinge of the ‘activation segment’ which keeps the enzyme constitutively active [8,9]. In Arab Berbers, rs34637584_A (p.G2019S) has a background frequency of 0.9% and accounts for >30% of sporadic patients and 40% of those with a family history of PD [10]. Despite the gene’s identification through linkage as a dominant Mendelian disorder [11], the penetrance (defined as the probability of the phenotype given the genotype) of rs34637584_A is incomplete [12]. Although subtle prodromal signs may be missed, including hyposmia, REM sleep behavior disorder, and orthostasis, the expressivity (defined as the variability in the presentation of clinical phenotypes) of *LRRK2* parkinsonism is as variable as idiopathic PD [13]. Conceivably, penetrance and expressivity are a function of other genetic and environmental modifiers. Notably, polymorphisms within the *LRRK2* locus are associated with PD susceptibility [14] and the age at onset (AOO) in progressive supranuclear palsy [15], and these may influence *LRRK2* expression, protein interactions, and kinase activation [16]. *LRRK2* is highly expressed in the cells of the innate immune system, including peripheral monocytes and macrophages, particularly B-cells, T-cells, and CD16+ monocytes [17]. *LRRK2* expression can also be induced in immune cells upon stimulation with classic pro-inflammatory bacterial protein-lipopolysaccharide (LPS) [18], as well as pathogen exposure [17]. Thus, it is plausible that penetrance may be influenced by *LRRK2*’s role in intracellular innate immunity and pathogen responses. In animal models, *LRRK2* p.G2019S confers a host survival advantage against infectious disease [19,20,21]. Notably, variability in the *LRRK2* locus is also associated with inflammatory bowel disease [22], pediatric immune disorders [23], and type-1 response in leprosy [24]. Here, we investigate the effects of genetic variability *in cis* and *in trans* of rs34637584_A in a sample from the Tunisian Arab-Berber population. We assess the relationship with the AOO and the genetic evidence for ancestral *LRRK2* haplotype selection.

## 2. Materials and Methods

### 2.1. Participants and Clinical Evaluation

A total of 755 individuals, 434 patients and 321 controls, were included in this study. Of these, 232 were *LRRK2* p.G2019S carriers, 220 of which were affected by PD (Table 1). All were of Tunisian descent, and family relationships were sought, and pedigrees were constructed when appropriate. All participants were aged 18 years or older at neurological assessment and provided written informed consent. Clinical exams were performed by movement disorder specialty-trained neurologists and diagnoses were made using the UK Brain Bank Criteria for PD [25], but inclusive of those with a family history. Blood samples were sourced ethically and their use in research was in accordance with the terms of the written informed consent. The instruments, selection criteria, and data collected on patients and control participants were comprehensive and have previously been published [13]. This study was approved by the ethics board of the Mongi Ben Hamada National Institute of Neurology and these data and analyses were reviewed and approved by the Institutional Review Board of the University of Florida.

### 2.2. Genotyping and Imputation

Individuals were genotyped using the Affymetrix 500K (ThermoFisher Scientific, Waltham, MA, USA) or Multi-Ethnic Genotyping Array (MEGA) (Illumina, San Diego, CA, USA). *LRRK2* p.G2019S (rs34637584_A) was genotyped using Taqman probe C 63498123_10 (ThermoFisher Scientific, Waltham, MA, USA). Chromosome 12 SNP imputation and haplotype phasing were carried out using a European genome reference with and without Tunisian WGS. As there are no Arab-Berber reference genomes within public databases, we selected 13 rs34637584_A heterozygotes with extreme AOO phenotypes (7 with young onset PD (mean AOO = 34.6 SD = 7.02 (22–42) years), and 6 elderly but clinically asymptomatic individuals (mean age = 78.7 SD = 7.0 (69–89) years) for whole-genome sequencing (WGS). SNPs were then filtered by genotyping rate (<95%), Hardy-Weinberg equilibrium (*p* < 0.01), R^2^ (<95%), and imputation accuracy (<98%) [26]. Imputed data were imported into and combined within PLINK [27]. Appropriate phasing was confirmed for the most distal 5′ and 3′ SNP alleles *in cis* by inspecting the same marker data within *LRRK2* p.G2019S pedigrees [28].

### 2.3. Phylogenic Analysis

Patients with idiopathic PD and individuals with rs34637584_A, regardless of disease status, were included in the phylogenetic analysis. Distinct haplotypes spanning the *LRRK2* p.G2019S mutation were identified in R using the ‘phangorn’ package [29,30] and used the Tamura and Nei ’93 models [31]. Estimation of the maximum likelihood of distinct haplotypes was performed with 1000 permutations. Linear mixed-effects regression analyses assessed *LRRK2* haplotype effects *in cis* or *in trans* on the age at onset (AOO), using the ‘coxme’ R package, adjusting for sex and kinship coefficients, as previously described [32]. A variable-length Markov chain Monte Carlo method within Beagle3.3 was used to extract haplotypes potentially associated with AOO, dichotomizing groups by their median and comparing quartile extremes [33].

### 2.4. Estimation of Generational Age

The age of *LRRK2* p.G2019S was estimated using marker allele frequencies and the recombination fraction between haplotypes. The age of the mutation in generations (g) was derived from g = ln(δ)/ln(1 − θ) for each marker [34]. Here, δ represents the linkage equilibrium index:

(P_m_ − P_n_)/(1 − P_n_) (1)

where P_m_ and P_n_ represent the allele frequency in carriers and noncarriers, respectively, and θ represents the recombination fraction as a function of the distance between the candidate location and the *LRRK2* gene [4].

### 2.5. Evaluation of Positive Selection

Integrated haplotype scores are a measure of selection based on the extended haplotype homozygosity statistic (EHH), as previously described [35]. This measures the decay of identity, as a function of distance, of haplotypes that carry a specified “core” allele at one end. For each allele, haplotype homozygosity starts at 1 and decays to 0 with increasing distance from the core site. When strongly selected, an allele may rapidly increase in frequency and will tend to have high levels of haplotype homozygosity extending further than expected under a neutral model. Hence, the integral of the observed decay of the EHH away from a specified marker, namely rs34637584, can be computed with respect to the ancestral (rs34637584_G) or derived core allele (rs34637584_A) until it reaches 0.05 and then summed in both directions, denoted as iHHA or iHHD. The test statistic iHS is given as

Unstandardized iHS = ln (iHHA/iHHD)(2)

When the rate of EHH decay is similar on the ancestral and derived alleles, iHHA/iHHD ≍1 and the unstandardized iHS is ≍0. Large negative values indicate unusually long haplotypes carrying the derived allele; large positive values indicate long haplotypes carrying the ancestral allele. Since, in neutral models, low-frequency alleles are generally younger and are associated with longer haplotypes than higher-frequency alleles, the unstandardized iHS is adjusted to obtain a final statistic which has a mean of 0 and a variance of 1, regardless of the allele frequency at the core SNP:

iHS = ln (iHHA/iHHD) − E_p_ [ln (iHHA/iHHD)]/SD_p_ [ln (iHHA/iHHD)] (3)

The expectation (E) and standard deviation (SD) of ln(iHHA/iHHD) are estimated from the empirical distribution at the SNPs, the derived allele frequency ‘p’ of which matches the frequency at the core SNP. The iHS is constructed to have an approximately standard normal distribution and, hence, the sizes of the iHS signals from different SNPs are directly comparable regardless of the allele frequencies at those SNPs. Since iHS is standardized using a chromosome 12-wide empirical distribution, it provides a measure of how unusual the haplotypes around a given SNP are, relative to chromosome 12 as a whole. Nevertheless, it does not provide a formal significance test, but that can be achieved by bootstrapping.

To examine the intervals of adaptation, we characterized the integrated haplotype scores (iHS) for two groups: (1) with rs34637584_A (*LRRK2* c.6055G>A (p.G2019S) including heterozygotes and homozygotes (AG and AA)) and (2) with rs34637584_G only (idiopathic PD and control subjects without the *LRRK2* p.G2019S mutation (GG)). SHAPEIT2 [36] and selscan v1.1.0 software calculated an unstandardized iHS, and the absolute value of the iHS scores was plotted against their genomic position.

To test the positive selection *in trans*, *p*-values were simulated by bootstrap sampling 100,000 iHSs across chromosome 12 for comparison. Positive selection was again tested after removing alleles with known inflammatory-associated variants within the *LRRK2* locus (Crohn’s disease: rs11175593_T and rs4768236_C; and Pediatric Autoimmunity: rs17466626_G). One or more of these SNPs was identified in 214 alleles, leaving 208 alleles.

## 3. Results

**Figure 1:**
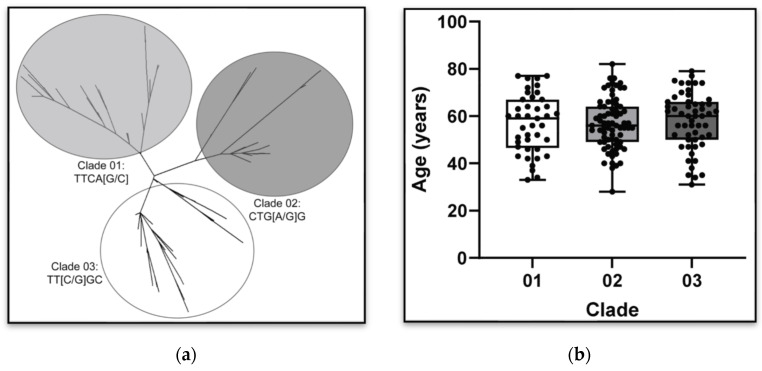
Three clades defined by the variability in 5 SNPs (rs2638245, rs10878199, rs2638271, rs2708438, and rs1388587) were identified within this sample. (**a**) An unrooted maximum likelihood phylogenic tree shows three prominent clades in this Tunisian cohort. These three clades are defined by: TTCA[G/C], n = 41, freq = 0.24; CTG[A/G]G, n = 73, freq = 0.43; and TT[C/G]GC, n = 54, freq = 0.32, respectively. (**b**) The age of initial symptom onset for affected heterozygotes in each of the three trans clades. No significant difference was observed (z = 0.40, *p* = 0.69).

The results for rs34637584_A were generated by direct genotyping and added to the high-density array data. Imputation yielded 16,997 SNPs on chromosome 12 (average minor allele frequency (MAF) = 0.25 ± 0.13 SD, range 0.50–0.017). Data from the *LRRK2* p.G2019S heterozygotes and homozygotes were compared to define allelic variability *in cis* for the longest, most parsimonious allele for the majority of samples. This spanned a genomic distance of 396 Kb, from rs878010 to rs73110066, and included a total of 69 markers in addition to the pathogenic *LRRK2* c.6055A variant (rs34637584_A at 12:40340400 (GRCh38)) (Supplementary Table S1). The 396 Kb haplotype *in cis* included complete genotyping data for all samples (n = 145) and was identical in all but one unaffected control with rs2404840_G>A, which might have been due to recombination. SNP frequencies in the most parsimonious *LRRK2* haplotype versus allele frequencies in unrelated control participants without rs34637584_A enabled the age of the mutation to be calculated as approximately 40 (95% CI 28–52) generations. Assuming 30 years per generation, the rs34637584_A ancestral allele in this sample originated approximately 1200 ± 360 years ago. Within the same dataset, we observed 81 alternate *LRRK2* haplotypes *in trans* (unique haplotypes defined as having a ≥1/69 difference in marker alleles). A variable-length Markov chain Monte Carlo method [33], implemented within Beagle3.3, was used to identify the shortest haplotype *in trans* most associated with AOO, but none were observed that reached significance after correction for multiple testing. Additionally, a maximum likelihood method was used to resolve haplotype relationships as a phylogenetic tree. This method identified three major clades from a central unrooted node and could be partitioned by five major SNPs (rs2638245, rs10878199, rs2638271, rs2708438, and rs1388587) that spanned the 40.1–40.3 Mb interval. Nevertheless, no clade association with AOO was apparent in this sample (z = 0.40, *p* = 0.69) (Figure 1).

**Figure 2:**
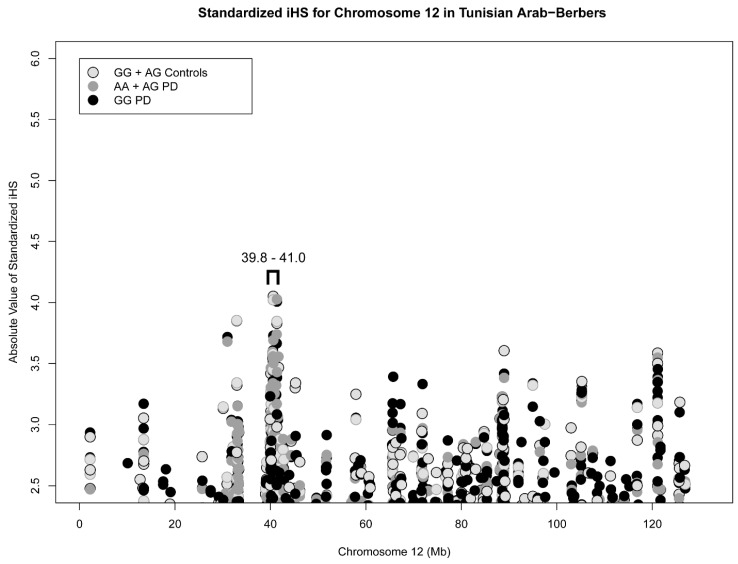
Absolute value of elevated iHSs > 2.5 across chromosome 12. A cluster of elevated iHSs can be observed within the 39.8–41.0 Mb range, which encompasses *LRRK2* p.G2019S haplotypes and is consistent with positive selection within the region. *LRRK2* p.G2019S is encoded by GRCh38 NC_000012.12:g.40340400G>A (‘A’ is the mutant allele, as boxed above).

Lastly, we investigated whether the background frequency of the highly conserved rs34637584_A haplotype might be driven by recent positive selection. Integrated haplotype scores (iHS) summarized the evidence for the entirety of chromosome 12, as illustrated for affected heterozygotes and homozygotes (AG+AA), and wild-type (GG) affected and unaffected individuals. A cluster of higher iHSs (>2.5) demarked an interval between 39.8 and 41.0 Mb (Figure 2). The distribution of iHS scores for the entirety of the chromosome 12 iHS values (minus the *LRRK2* locus) was bootstrapped and suggested that this cluster was highly significant compared to scores for the rest of the chromosome (*p* = 4.50 × 10^−18^). Curiously, iHS scores within the *LRRK2* locus from 40.2 to 41.0 Mb were nominally significant for rs34637584_G wild-type alleles (GG_all_ = 422, *p* = 2.95 × 10^−4^ to 1.61 × 10^−6^. Table 2). As several inflammatory disorders are associated with the *LRRK2* locus, we removed any individual with these disease-associated SNP alleles, namely rs11175593_T [22], rs4768236_C [37], and/or rs17466626_G [23] (GG_nim_ = 208), and the *LRRK2* signal was ablated (Table 2). Despite the reduction in sample size, the mean iHS scores and their distributions were comparable in sub-groups with and without inflammatory markers (0.79 ± 0.59 SD vs. 0.80 ± 0.59 SD) (Supplementary Figure S1). Overall, these results were consistent with positive evolutionary selection for the *LRRK2* region, not just for the rs34637584_A allele.

## 4. Discussion

This study supports and extends prior studies suggesting that *LRRK2* p.G2019S heterozygotes are descendants of a common ancestral founder that originated at least 40 (95% CI 28–52) generations ago (Supplementary Table S1). This result is within the confidence interval of prior estimates (Supplementary Tables S1 and S2) [38,39,40,41,42,43,44]. The *LRRK2* locus includes SNPs that nominate genome-wide associations to several inflammatory disorders (Crohn’s disease [rs11175593_T] [22], inflammatory bowel disease [rs4768236_C] [37], pediatric immune diseases [rs17466626_G] [23], and platelet count [rs529898481_G] [45]). However, in our data, those alleles are not in linkage disequilibrium with rs34637584_A (the *LRRK2* c.6055A haplotype, all pairwise R^2^ values < 0.05). Rather, those alleles are captured on haplotypes *in trans*. Whether these variants confer a functional change in *LRRK2* expression or activity has yet to be demonstrated.

Haplotype phasing and imputation, especially from one population to another, can be quite inaccurate, given different patterns of linkage disequilibrium and allele frequencies [46]. Here, we used whole-genome sequencing from European references with/without a relatively small sample of whole genomes from Tunisia. Nevertheless, the imputation from these references was entirely consistent with the polymorphic alleles that were array-genotyped, and the haplotype phase was confirmed for accuracy in Tunisian pedigrees and Lrrk2 p.G2019S homozygotes.

Despite our limited sample size, the cluster of iHS values around the *LRRK2* locus was indicative of positive selection for *LRRK2* rs34637584_A. Although Tunisia has a high frequency of consanguineous marriages, neither isolation nor genetic drift are likely to produce the distribution of values observed. Overall, the burden of evidence from our data and others suggests that rs34637584_A, and the constitutive *LRRK2* kinase activity it confers, offers a survival advantage to reproductive age. To date, this has enabled a >19-fold increase in the background frequency of rs34637584_A in Tunisia, in our sample, compared to the global mean (rs34637584_A MAF_Tunisia_ = 0.0094 (7/742) [10]; MAF_gnomADr2.1-all_ = 0.0004884 (138/282,542), _gnomADr2.1-African_ = 0.0001202 (3/24,962). Fisher’s *p* = 9.53 × 10^−27^).

Although the evolutionary forces driving positive selection for *LRRK2* alleles are unknown, epidemiologic and experimental research on pathogens restricted by *LRRK2* kinase activity may be informative [17,21]. In this regard, it is worth noting that the frequency of *GBA* variants, that are also associated with PD, has largely been driven by enhanced survival against tuberculosis [47]. Peripheral and central immune mechanisms that contribute to PD have yet to be proven [48] but from a genetic perspective *LRRK2* variability is clearly associated with multiple immune-related disorders [49,50,51,52]. Healthy individuals carrying the *LRRK2* p.G2019S mutation show increased levels of pro-inflammatory cytokines in serum and, in sporadic PD patients, *LRRK2* levels are elevated in neutrophils, B-cells, T-cells, and CD16+ monocytes, when compared to healthy controls [53,54].

Multiple interactors of *LRRK2* have been discovered including a number of Rab GTPase substrates, such as RAB8, RAB10, RAB12 and RAB29, that may further induce *LRRK2* membrane recruitment, kinase activation and phosphorylation [53,55]. However, only RAB32 p.S71R, that constitutively increases *LRRK2* kinase activation, is genetically linked and associated with PD [56]. RAB32 is important in autophagosome recycling, and in the biogenesis and transport of melanosomes in melanocytes, and similar molecular components are necessary for catecholamine metabolism and pigment production in the *substantia nigra* [56]. RAB32 also traffics mitochondrially derived itaconic acid to the pathogen-containing vacuole to inhibit bacterial growth [57]. Curiously, it also interacts with PINK1 [56], which instigates mitophagy, and for which loss-of-function mutations are best described in Tunisian families with parkinsonism [58]. Similarly, VPS35 p.D620N, a core component of the retromer, is genetically linked to PD and activates *LRRK2* kinase [59,60]. Mechanistically, retromer is also central to the innate immune response and often corrupted by intracellular pathogens [61].

The ability to demonstrate positive selection for other linked loci that cause PD is currently limited by sample size. Nevertheless, most Mendelian gene mutations that cause PD impinge on phagolysosome biology and/or intracellular innate immunity, and their origin appears to illustrate a convergent evolution. It is now crucial to consider how peripheral and central immunity influences the vulnerability of dopaminergic neurons in *LRRK2* and idiopathic PD, in which both cell-autonomous [62] and non-autonomous mechanisms evidently contribute [20].

## Figures and Tables

**Table 1:** Demographics of *LRRK2* p.G2019S and idiopathic patients.

	*LRRK2* Wild Type		*LRRK2* p.G2019S	
	Patients	Controls	Patients	Controls
N	214	321	220	12
Number of men (%)	105 (49.1%)	168 (52.5%)	124 (56%)	6 (50%)
Mean age (SD)	68.1 (12.8)	62.4 (11)	67.6 (12.6)	56.7 (10.9)
Median age (IQR)	68 (59–76)	62 (53–69)	69 (48–90)	54.5 (38–72)
Mean age of onset (SD)	54.9 (14.5)	-	57.1 (11.6)	-
Median age at onset (IQR)	58 (46–66)	-	57 (40–74)	-

**Table 2:** Evidence for positive selection in *LRRK2* wild-type alleles with and without inflammatory markers.

*p*-Value (w/ Inflammatory Markers)	*p*-Value (w/out Inflammatory Markers) *	Window Bin [Start–End (Mb)]
0.16	0.22	39.6–39.85
5.56 × 10^−5^	0.011	39.8–40.05
**1.61 × 10^−6^**	**0.27**	**40.0–40.25**
**3.64 × 10^−4^**	**0.32**	**40.2–40.45**
2.95 × 10^−4^	0.14	40.4–40.65
0.36	0.27	40.6–40.85
0.55	0.08	40.8–41.05
0.45	0.99	41.0–41.25
0.0060	0.0058	41.2–41.45
0.34	0.58	41.4–41.65
0.06	0.15	41.6–41.85
1.28 × 10^−4^	5.71 × 10^−5^	41.8–42.05
0.093	0.12	42.0–42.25
0.26	0.04	42.2–42.45
0.61	0.53	42.4–42.65
0.51	0.34	42.6–44.85

* Inflammatory SNP alleles considered were rs11175593_T^13^, rs4768236_C^17^, and/or rs17466626._G^14^. In bold are *p*-values < 0.05 for windows including the *LRRK2* locus chr12:40224997-40369284 (GRCh38, NM_198578).

## Data Availability

The data presented in this study are available on request from the corresponding author due to privacy and ethical restrictions.

## Supplementary Materials

The following supporting information can be downloaded at https://www.mdpi.com/article/10.3390/genes15070878/s1, Figure S1: Integrated haplotype scores in *LRRK2* c.6055G (rs34637584_A) samples with and without inflammatory risk markers; Table S1: Analysis of mutational age; Table S2: Published age estimates of *LRRK2* c.6055A (rs34637584_A).
