# Supplementary material for: The Evolution of Genetic Variability at the LRRK2 Locus
Source: Genes (Basel). 2024 Jul 3;15(7):878. doi: 10.3390/genes15070878 (PMC11275506; doi:10.3390/genes15070878)
Supplement: Supplementary file 1 [file genes-15-00878-s001.zip › GENES_SupplementaryTable02_DTG_V02_LRRK2-Selection.pdf]

Supplementary Table S2: Published age estimates of LRRK2 c.6055A (rs34637584_A)

Study	Genotyping Markers (N)	Origin	Age Estimates (years (95% CI))
Lesage et al. 2005[38]	Microsatellites (17)<br>SNPs (4)	Eastern European and North America	725<br>(375 – 1375)
Zabetian et al. 2006[39]	Microsatellites (13)<br>SNPs (12)	European and Jewish Ancestry	1875<br>(1375 – 2600)
Warren et al. 2008[40]	Microsatellites (2)<br>SNPs (39)	North African	2600<br>(1950 – 3850)
Bar-Shira et al. 2009[41]	Microsatellites (15)<br>SNPs (1)	Ashkenazi Jew	1525<br>(1300 – 1800)
Lesage et al. 2010[42]	Microsatellites (20)<br>SNPs (54)	North African, European, and Jewish Ancestry	4000<br>(2925 – 6250)
Lucotte et al. 2012[43]	Microsatellites (7)<br>SNPs (1)	French, Spanish, North African, and Sephardi Jewish	3840<br>(3210-5400)
Ben El Haj et al. 2017[44]	Microsatellites (10)	North African Arab Berbers	5000<br>(3075 – 8700)
This work	SNPs (69)	North African Arab Berbers	1200<br>(840 – 1560)
