# Supplementary material for: The Evolution of Genetic Variability at the LRRK2 Locus
Source: Genes (Basel). 2024 Jul 3;15(7):878. doi: 10.3390/genes15070878 (PMC11275506; doi:10.3390/genes15070878)
Supplement: Supplementary file 1 [file genes-15-00878-s001.zip › GENES_SupplementaryTable01_DTG_V01_Lrrk2-Selection.pdf]

Supplementary Table S1: Analysis of mutational age

SNP	Mutation	Mb (GRCh38)	P_n (freq in non-carriers)	P_m (freq in carriers)	Theta	Delta	Estimated Age of Mutation (generations)
rs878010	A/T	40.108843	0.352	0.657	0.135581	0.470679	5.17
rs900253	C/T	40.108943	0.361	0.664	0.135477	0.474178	5.13
rs1482287	A/T	40.113336	0.375	0.667	0.134137	0.4672	5.28
rs7310430	A/T	40.114388	0.366	0.664	0.134012	0.470032	5.25
rs4533099	A/C	40.127691	0.134	0.08	0.12672	0.062356	20.48
rs11564137	C/T	40.127785	0.176	0.07	0.126687	0.128641	15.14
rs11175260	C/T	40.128094	0.063	0.04	0.126587	0.024546	27.39
rs10784386	A/G	40.135842	0.382	0.67	0.123839	0.466019	5.78
rs2404574	C/T	40.138199	0.186	0.073	0.122652	0.138821	15.09
rs767940	C/G	40.138693	0.241	0.153	0.122392	0.115942	16.50
rs10878123	A/T	40.138731	0.063	0.039	0.12237	0.025614	28.07
rs726330	A/C	40.142259	0.265	0.105	0.120226	0.217687	11.90
rs2638271	C/G	40.147232	0.316	0.151	0.117376	0.241228	11.39
rs2638270	G/T	40.147356	0.26	0.122	0.117308	0.186486	13.46
rs11564122	A/G	40.153948	0.061	0.018	0.112092	0.045793	25.94
rs11564215	A/C	40.156271	0.109	0.072	0.111038	0.041526	27.03
rs11564157	C/T	40.160295	0.136	0.089	0.108082	0.054398	25.45
rs1388587	C/G	40.167733	0.359	0.226	0.103539	0.207488	14.39
rs11175456	A/G	40.173779	0.142	0.091	0.100482	0.059441	26.66
rs10878199	G/T	40.173801	0.232	0.14	0.100458	0.119792	20.04
rs2638245	C/T	40.178345	0.405	0.683	0.096405	0.467227	7.51
rs2708404	A/G	40.181828	0.25	0.099	0.094713	0.201333	16.11
rs2046932	A/G	40.186638	0.087	0.032	0.093596	0.060241	28.59
rs1491924	C/T	40.194128	0.066	0.042	0.091636	0.025696	38.10
rs11564274	C/T	40.196818	0.072	0.027	0.08961	0.048491	32.24
rs2723266	C/T	40.201740	0.022	0.002	0.08253	0.02045	45.16
rs1491940	A/T	40.204542	0.476	0.253	0.082318	0.425573	9.95
rs2723261	C/T	40.208006	0.478	0.253	0.08197	0.431034	9.84
rs11175593	C/T	40.208138	0.05	0.033	0.08196	0.017895	47.05
rs2708437	C/T	40.220977	0.091	0.058	0.076446	0.036304	41.70
rs2708438	A/G	40.221630	0.091	0.058	0.076182	0.036304	41.85
rs1491943	A/T	40.226614	0.044	0.029	0.073563	0.01569	54.37
rs1352878	C/T	40.232483	0.027	0.012	0.071417	0.015416	56.31
rs2723267	A/C	40.232887	0.017	0.005	0.071294	0.012208	59.57
rs2249281	C/T	40.237556	0.037	0.012	0.068101	0.025961	51.77
rs10878244	A/G	40.237806	0.099	0.016	0.067702	0.09212	34.02

rs10878245	C/T	40.237989	0.422	0.701	0.067137	0.482699	10.48
rs7134379	C/T	40.251072	0.347	0.167	0.06087	0.275651	20.52
rs4293189	A/G	40.263423	0.324	0.667	0.045654	0.507396	14.52
rs4768224	A/T	40.267601	0.348	0.153	0.043791	0.29908	26.96
rs10878356	A/G	40.316155	0.232	0.101	0.025452	0.170573	68.60
rs11564177	C/T	40.320530	0.114	0.026	0.023904	0.099323	95.45
rs10878368	A/T	40.320983	0.49	0.746	0.023893	0.501961	28.50
rs10506153	C/T	40.331769	0.119	0.028	0.012793	0.103292	176.32
rs34637584	G/A	40.734202	-	-	-	-	-
rs11564259	C/G	40.398395	0.052	0.035	0.024735	0.017932	160.55
rs11564172	A/C	40.398422	0.103	0.043	0.024736	0.06689	107.99
rs11564258	A/G	40.398498	0.052	0.035	0.024738	0.017932	160.53
rs971616	G/T	40.399054	0.054	0.035	0.024755	0.020085	155.90
rs1427261	C/T	40.404756	0.127	0.046	0.026218	0.092784	89.49
rs2404840	A/G	40.409986	0.452	0.722	0.027056	0.492701	25.81
rs6581723	A/G	40.410584	0.132	0.047	0.027144	0.097926	84.43
rs11564144	A/C	40.424193	0.054	0.037	0.050544	0.01797	77.49
rs11564249	C/T	40.426601	0.052	0.035	0.051238	0.017932	76.45
rs11564247	C/T	40.427676	0.052	0.035	0.051962	0.017932	75.36
rs11564168	C/T	40.436781	0.201	0.089	0.077609	0.140175	24.32
rs11564234	G/T	40.437044	0.114	0.031	0.07898	0.093679	28.78
rs11564233	A/G	40.437326	0.472	0.784	0.080103	0.590909	6.30
rs10878583	C/G	40.437793	0.472	0.788	0.081147	0.598485	6.07
rs3912855	A/G	40.455671	0.088	0.037	0.086732	0.055921	31.79
rs17128239	A/C	40.460638	0.001	0.002	0.087862	0.001001	75.10
rs17128301	A/G	40.479054	0.109	0.03	0.091534	0.088664	25.24
rs2588400	C/T	40.479449	0.204	0.094	0.091995	0.138191	20.51
rs11176811	A/T	40.479476	0.196	0.085	0.091995	0.13806	20.52
rs7975790	G/T	40.498527	0.193	0.085	0.09467	0.133829	20.22
rs1444216	A/T	40.498716	0.203	0.094	0.094703	0.136763	20.00
rs11176884	C/T	40.499572	0.196	0.085	0.094862	0.13806	19.87
rs4767978	C/G	40.499694	0.274	0.684	0.094886	0.564738	5.73
rs2197356	A/G	40.500186	0.086	0.032	0.094988	0.059081	28.34
rs73110066	C/T	40.505059	0.085	0.037	0.100832	0.052459	27.736
