# Supplementary material for: The Evolution of Genetic Variability at the LRRK2 Locus
Source: Genes (Basel). 2024 Jul 3;15(7):878. doi: 10.3390/genes15070878 (PMC11275506; doi:10.3390/genes15070878)
Supplement: Supplementary file 1 [file genes-15-00878-s001.zip › GENES_SupplementaryFigure01_DTG_V02_LRRK2-Selection.pdf]

Supplementary Figure S1: Integrated haplotype scores in LRRK2 c.6055G (rs34637584_A) samples, with and without inflammatory risk markers

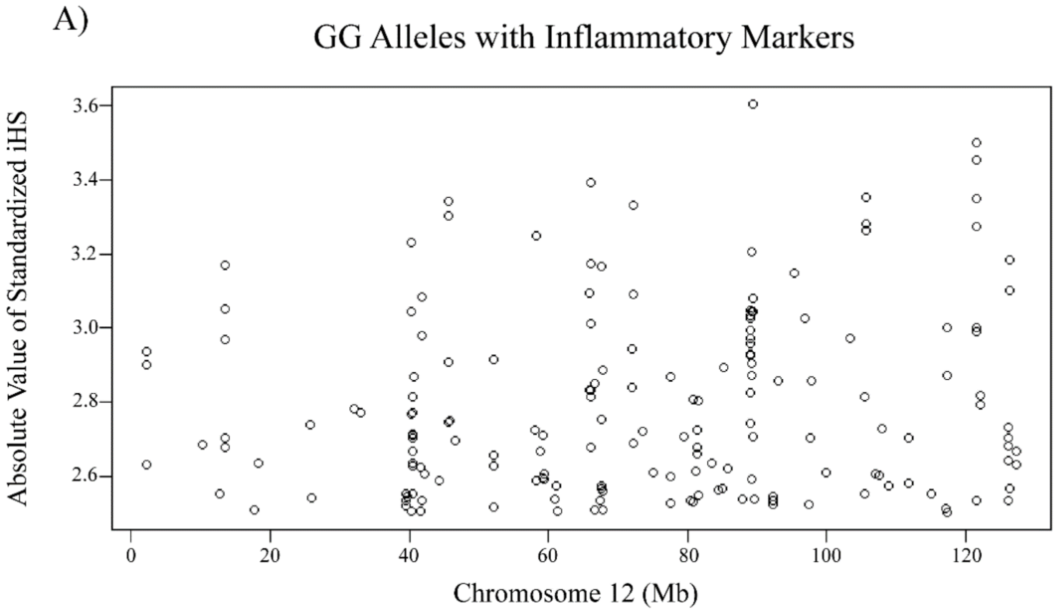

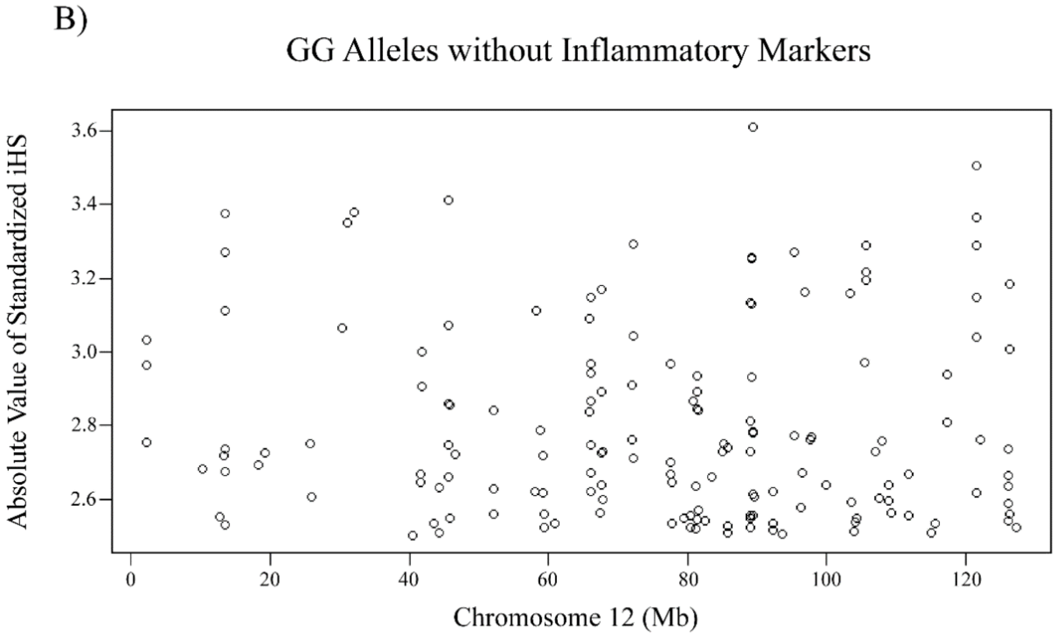

Absolute value of standardized integrated haplotype scores across chromosome 12 for: A) homozygous wildtype individuals with, and B) without alleles for known inflammatory risk markers within the *LRRK2* locus. A small cluster of elevated iHS values can be observed in the group where risk alleles remain, but the signal is lost when they are removed.
